# Genetic Influences on Alcoholism Risk: A Review of Adoption and Twin Studies

**Published:** 1995

**Authors:** Andrew C. Heath

**Affiliations:** Andrew C. Heath, D.Phil., is an associate professor in the Department of Psychiatry, Washington University School of Medicine, St. Louis, Missouri

**Keywords:** adoption study, twin study, AOD dependence, risk factors, hereditary factors, environmental factors, biological family relation, adoptive family relation, research and evaluation method, gender differences

## Abstract

Researchers have used two different strategies to determine the relative contributions of genes and shared environment to the development of alcoholism among family members: the adoption study and the twin study. Adoption studies compare the risk to biological relatives with the risk to adoptive relatives of alcoholics. Twin studies compare identical and fraternal pairs of twins reared in the same environment. Evidence indicates an important genetic influence on alcoholism risk; this influence appears as strong in women as in men. These results have been remarkably consistent despite major differences between research methods.

The tendency for alcoholism to run in families has long been recognized through controlled family studies beginning in the 1950’s (Amark 1951; Bleuler 1955; Cotton 1979). Research findings have indicated that alcoholism can arise in families through genetic or environmental causes or through a mixture of both. Researchers have used two different strategies for determining the proportional contributions of genes and shared family environment to the development of alcoholism among family members: the adoption study and the twin study. The adoption study compares the risk of alcoholism in biological relatives with the risk in adoptive relatives of alcoholics (e.g., an adopted-away child of an alcoholic parent). In contrast, the twin study compares the risk of alcoholism in identical and fraternal pairs of twins reared in the same environment. Although studies of the genetic aspects of alcoholism have expanded to include molecular and animal studies, it is helpful to reexamine adoption and twin evidence; both types of data are important bases for subsequent research on genetic influences on alcoholism. This article briefly summarizes and updates a recent review reanalyzing the major published studies on gender differences in the genetic contribution to alcoholism risk (Heath et al. in press). (For more information on the methodology of these types of research, see the related articles in the Tools of Genetic Research section, pp. 190–227.)

This article focuses on studies that have systematically used samples ascertained from birth or adoption records. Unfortunately, however, this review excludes several important studies (Gurling et al. 1984; Pickens et al. 1991; Caldwell and Gottesman 1991; McGue et al. 1992; Heath et al. 1994), because various technical issues place those studies beyond the scope of this review (for further details, see Heath et al. in press).

## Adoption Study Findings

### A Pioneering Study

The first adoption-study evidence for an important genetic contribution to alcoholism risk was produced in Scandinavia. In Copenhagen, Denmark, Goodwin and colleagues (1973, 1974, 1977*a*,*b*) used official registries to identify biological parents who had histories of alcoholism and who had given up a child for early adoption by nonrelatives. The researchers used biological parents who had no known histories of alcoholism but who also had given up a child for early adoption as control subjects. Interviews were conducted with adult sons and daughters of both groups to determine the prevalence of alcoholism among them. The researchers speculated that if the genetic contribution to alcoholism were important, the rates of alcoholism should be higher in the adopted-away offspring of the alcoholic biological parents than in the adopted-away offspring of the control parents.

The investigators also identified a subset of biological parents who had given up one child for adoption but had reared a second child themselves. This subset could demonstrate the effects of environment on the children. If growing up in the environment of an alcoholic parent contributes significantly to alcoholism risk, this risk should be higher in the nonadopted sons and daughters of alcoholics, compared with the adopted-away sons and daughters.

According to the findings, 8.9 percent of the fathers and 1.6 percent of the mothers who gave their offspring up for adoption had been hospitalized for alcoholism. Heath and colleagues (in press) estimated that the proportion of all adults in the population of Copenhagen who were in the same age range as the biological parents and who had been hospitalized for alcoholism at some stage in their lives was 2 percent for men and 0.5 percent for women. These figures are almost certainly overestimates. Compared with what would be expected for the population as a whole, the lifetime prevalence of hospitalization for alcoholism is at least four times higher in the biological fathers and three times higher in the biological mothers of the children who were given up for adoption. Therefore, if alcoholism is genetically influenced, then adoptees as a group would be at higher risk than the general population and would have elevated rates of alcoholism. The higher genetic risk among adoptees is a recurrent finding in the major adoption studies and should be taken into consideration when analyzing results.

Goodwin and colleagues (1973, 1977*a*,*b*) reported an estimated risk ratio of 3.6 for adopted-away sons of alcoholics and 3.4 for nonadopted sons of alcoholics, compared with an assigned risk value of 1.0 for control adoptees.^1^ Likewise, only 2 percent of adopted-away daughters of alcoholics and 3 percent of nonadopted daughters were diagnosed as having histories of alcoholism, compared with 4 percent of control adoptees, giving risk ratios of only 0.5 and 0.75. Risk ratios using estimates of lifetime alcoholism prevalence in the general population are unchanged in men but become 2.0 and 3.0 for adopted and non-adopted daughters of alcoholics based on Goodwin’s estimated 1-percent prevalence for alcoholism in women. According to these findings, rates of alcoholism are significantly elevated in both the adopted and nonadopted sons of alcoholics, results which are consistent with a genetic influence on alcoholism risk in men. Results for women are not significant.

What can we conclude from these findings? First, it appears that there is a genetic influence on alcoholism in men. The Copenhagen data do not support a firm conclusion with respect to a genetic influence in women. These data also do not provide evidence for a significant environmental impact of parental alcoholism, because the risk to nonadopted offspring is no greater than the risk to adopted offspring, although moderately strong environmental influences could remain undetected given the sample sizes used.

A limitation of the Copenhagen study is that no direct interviews were conducted with adoptive parents; therefore, the possibility that selective placement occurred, leading to an above-average probability that the child of an alcoholic biological parent would be raised by an alcoholic adoptive parent, cannot be completely ruled out. Such selective placement would cause the importance of genetic effects to be overestimated. It seems implausible, however, that this effect could completely explain the elevated risk to adopted-away sons of alcoholic parents, since their risk is no less than that to nonadopted sons of alcoholics. Family history reports by the adoptees suggest that, if anything, alcohol problems were less prevalent in the adoptive fathers of the adoptees who were sons of alcoholics (12 percent) than in the control adoptees (22 percent), a result which also suggests that selective placement of adoptees could not explain these findings.

### More Recent Adoption Studies

#### Sweden

A separate adoption study conducted in Scandinavia (Bohman et al. 1981; Cloninger et al. 1981, 1985) replicated the Copenhagen study findings using different procedures. No direct interviews with adoptees were conducted. Prevalence of alcoholism was estimated from records of the Stockholm Temperance Board. Records were obtained for both biological parents and their adopted-away offspring. Although additional information was available from other agencies (e.g., welfare reports and national health insurance records) and was used in some articles by Cloninger and colleagues, the data reanalyzed here are limited to Temperance Board registration data as reported by Cloninger and colleagues (1985).

By using archival records, the Stockholm study was able to obtain data on the entire sample of adoptees. Thus, prevalence rates for alcoholism are available for the total sample of biological parents and adoptees. Significant associations are found between Temperance Board registrations for biological fathers and their adopted-away sons (i.e., a risk ratio of 1.3) and for biological mothers and their adopted-away daughters (i.e., a risk ratio of 2.9). These ratios are consistent with a genetic contribution to alcoholism risk. However, the risk ratios for opposite-sex pair comparisons (i.e., mother-son and father-daughter pairs), although greater than one, are not statistically significant.

Cloninger and colleagues (1985) reported no significant association between adoptee alcoholism and Temperance Board registration in the adoptive parents. However, one cannot conclude from this finding that rearing environment in general has little impact on alcoholism risk. In less than 5 percent of adoptive families did either parent have a Temperance Board registration, implying that adoptees were being placed in low-risk environments.

#### United States

The only recent United States adoption studies on alcoholism for which results have been published are those conducted by Cadoret in Iowa. The fact that only one investigator has been able to conduct such studies may reflect the high degree of tenacity required to overcome State privacy regulations restricting access to information about the biological families of adoptees. In his earliest studies, Cadoret studied samples from Lutheran Social Services (LSS) (Cadoret et al. 1985; Cadoret 1994) and Iowa Children and Family Services (CFS) (Cadoret 1994; Cadoret et al. 1987). More recently, he has also studied samples recruited from a series of Catholic Adoption Agencies (CAA) (Cadoret 1994; Cadoret et al. 1995; Cutrona et al. 1994) and from four other adoption agencies (Cadoret 1994).

Original publications on the LSS and CFS samples are not always easy to interpret, as three types of control populations were used: adoptees with antisocial behavior in biological family members (usually, but not exclusively, parents),^2^ adoptees with alcohol problems in biological family members, and control adoptees. Results were reported for alcoholic versus nonalcoholic and for antisocial versus nonantisocial biological backgrounds. A subsequent publication (Cadoret 1994) has reported numbers for the alcoholic biological background versus control comparisons for males, and those numbers are used here; however, no similar breakdown appears to have been published for female adoptees. Thus, the “control” sample of female adoptees for each study includes adoptees from a nonalcoholic but antisocial biological background. Insofar as an overlap exists between genetic risk factors for alcoholism and antisocial behavior, this overlap will cause the risk ratios for women to be underestimated.

The two earliest Iowa adoption studies (i.e., the LSS and CFS) show significantly elevated risk to adopted-away sons from alcoholic biological backgrounds compared with control adoptees (i.e., risk ratios of 3.5 and 3.6, respectively), consistent with a genetic influence on alcoholism risk in men. For male adoptees in the remaining two samples, the risk to those from an alcoholic background is not significantly higher than that for control adoptees. In these latter studies, however, the rates of alcoholism are high, even in the control adoptees (from 55 to 58 percent), raising the possibility that the entire sample of adoptees, on average, came from high-risk biological backgrounds. Postulating a 28-percent prevalence rate for alcohol problems in the general population, the risk of alcoholism in adopted-away sons from alcoholic backgrounds is significantly greater than that for the general population.

Data from the LSS and CFS studies also allow us to examine the association between alcohol problems in the adoptive family and the occurrence of alcoholism in the adoptee. In each case, a significantly elevated alcoholism risk was found for male adoptees (i.e., risk ratios of 2.7 in the LSS study and 2.1 in the CFS study) raised in adoptive families in which at least one member experienced alcohol problems, compared with male adoptees raised in adoptive families in which no one else experienced alcohol problems. It should be noted, however, that because alcohol problems in the adoptive families could include problems experienced by siblings, analysis could overestimate the influence of the adoptive parents on the adoptees’ outcomes.

For female adoptees, data are available from both the LSS and CAA studies. Alcoholism in the biological background was associated with an increased risk of alcoholism in the LSS study but with a reduced risk in the CAA study. However, a high rate of alcoholism in female control adoptees in the latter study (28 percent) suggests that a similar problem exists to that observed for males from this source, with a population of adoptees that is at high risk for developing alcoholism. Compared with an estimated prevalence of 10 percent for female alcoholism, the rate of alcoholism in adopted-away daughters of alcoholics still is significantly elevated.

## Twin Studies

Twin studies of alcoholism have examined the increased risk of developing alcoholism in identical (i.e., monozygotic, or MZ) twins of alcoholics (who share the same genetic makeup as their alcoholic twins) and in fraternal (i.e., dizygotic, or DZ) twins of alcoholics (who, on average, share only half their genes) compared with the general population. If genetic influences or environmental factors shared by twin pairs growing up together are important, the percentage of twins of alcoholics with a current or past history of alcoholism should be much higher than the percentage in the general population. If genetic influences, in particular, are important, a significantly higher risk ratio should occur in MZ compared with DZ twin pairs.

### The Initial Study

The first twin study of alcoholism was conducted in Sweden in the 1950’s by Kaij (1960). This study used birth records and Temperance Board registration data to identify alcoholic male twins from Skåne, Sweden. Kaij conducted followup interviews with alcoholic twins and their co-twins,^3^ showing that twins having at least one Temperance Board registration exhibited a fivefold increase in probability of being diagnosed as alcoholic, thereby confirming the validity of registrations as a measure of alcohol problems. He also noted, however, that those twins with social problems were likely to be overrepresented among Temperance Board registrants.

Using as a criterion the requirement of at least one Temperance Board registration, the proportion of registered twins whose co-twins were also registered was significantly higher for the MZ than for the DZ pairs. Approximately 61 percent of MZ co-twins of twins with an alcohol problem and a significantly lower fraction (39 percent) of DZ co-twins of twins with an alcohol problem were registered. Based on national data, Kaij (1960) reported a lifetime alcoholism prevalence of 7.7 percent for males in the general population. Using this value gives a risk ratio of 9.1 for male MZ twins of registered co-twins and 6.2 for DZ co-twins. Again, these data are consistent with a genetic influence on alcoholism risk.

### Other Twin Studies

Subsequent studies using samples ascertained from birth records have confirmed, without exception, a higher risk to MZ compared with DZ twins of alcoholics, although this difference has not always been significant. Hrubec and Omenn (1981) identified alcoholism cases in a followup of a series of male same-sex twin pairs born between 1917 and 1927, identified originally from birth records, in which both twins engaged in military service during World War II. The researchers reviewed Veterans Administration (VA) medical records of approximately 13,486 male twin pairs, all of whom were age 50 at the time of the record review, to identify cases of alcoholism or alcoholic psychosis. Only 2.6 percent of MZ twins and 3.1 percent of DZ twins were reported as having any treatment history for alcohol problems. If one twin had a history of alcohol problems recorded, however, the probability that his or her twin also did was 26.3 percent for MZ twins (i.e., a risk ratio of 10.0) and 11.9 percent for DZ twins (i.e., a risk ratio of 3.8). These risk-ratio differences were highly statistically significant.

Three studies in Scandinavia have matched twin registries to national databases containing hospital discharge data. In Finland, Koskenvuo and colleagues (1984) conducted such a match using only an alcoholism discharge code and found a significantly higher risk ratio for male MZ than for male DZ twins of males hospitalized for alcoholism (i.e., 11.8 versus 5.5). Romanov and colleagues (1991) conducted a further followup of a subsample of the same twin cohort using a broader definition of alcoholism that included alcohol abuse-related discharge codes (e.g., alcoholic liver cirrhosis and alcoholic psychosis) and found a nonsignificant trend in the same direction (i.e., risk ratios of 8.8 versus 4.6, respectively). In Sweden, Allgulander and colleagues (1991, 1992) performed a similar match of the Swedish twin registry to alcohol-related discharge codes; these data again showed an elevated risk ratio in MZ, compared with DZ, male twins (i.e., 7.9 versus 5.3), although again the difference was not significant. For women, Koskenvuo and colleagues (1984) found no same-sex twin pairs in which both twins had an alcoholism discharge code, making it impossible to estimate a risk ratio. Allgulander and colleagues (1991, 1992) found substantial risk ratios for MZ and DZ female twins of female alcoholics (i.e., 41.9 and 16.5, respectively), but again these do not differ significantly.

The only other study to use a sample of twin pairs identified from birth records (Kendler et al. 1992) included only female same-sex pairs.^4^ Alcoholism was assessed using standard diagnostic criteria as defined by the *Diagnostic and Statistical Manual of Mental Disorders, Third Edition, Revised* (DSM–III–R) (American Psychiatric Association 1987). The relatively high lifetime prevalence rates of alcohol dependence (i.e., 10.2 percent for DZ twins and 8.1 percent for MZ twins) are consistent with what has been reported for a national probability sample in the National Co-morbidity Survey (Kessler et al. 1994).^5^ As in any survey of a general community sample, the majority of cases may be expected to be mild (for example, see Heath et al. 1994). Once again, however, rates of alcoholism were significantly elevated in both MZ and DZ twins of alcoholics (i.e., risk ratios of 3.9 and 2.4, respectively), and evidence showed a higher risk to MZ than to DZ twins (although this result did not reach statistical significance).^6^

## Combining Results From Different Studies

How can results across different studies or even within studies (e.g., between men and women) be compared? Risk-ratio estimates cannot simply be pooled because of differences in the estimated rates of alcoholism. Estimates of the prevalence of alcoholism are highly variable, depending on how alcoholism is defined. Thus, Kendler and colleagues (1992) reported a lifetime prevalence of alcohol dependence defined by DSM–III–R of 8 to 10 percent in women, whereas Hrubec and Omenn (1981) reported a prevalence in men of alcoholism treated through the VA system of only 2.6 to 3.1 percent. Estimates of risk ratios for relatives of alcoholics, which express risk to relatives as a ratio of the risk in the general population, are similarly variable.

One approach for comparing studies of disorders having a complex mode of inheritance has been a liability, or “threshold,” model. In this model, a person’s liability to develop alcoholism is assumed to be determined by the combined effects of many separate risk factors—genetic, environmental, or both. The distribution of liability to alcoholism in the general population is assumed to be continuous and to follow a bell curve. The majority of people exhibit an intermediate risk; some, a very low risk; and some, a very high risk. The model assumes that those whose liability exceeds some critical value (i.e., threshold) will become alcoholic. Changing the definition of alcoholism merely shifts the threshold to the right (i.e., fewer but more severe cases) or to the left (i.e., more but less severe cases). (For further discussion of the liability model in twin studies, see side-bar by Prescott and Kendler, pp. 204–205).

The liability model provides a natural framework for combining data from different studies using widely different definitions of alcoholism. It also allows us to compare the importance of genetic and/or shared environmental influences on alcoholism risk in men and women, despite the significant gender differences in the prevalence of alcoholism. In genetic studies, these liability correlations are usually expressed in terms of the causes of variation in alcoholism liability. Here, a simplified model is used that allows for the contributions of genes, family environment, and nonshared environmental experiences and ignores such complications as gene-environment correlation (i.e., the tendency of people at high genetic risk to be exposed also to high-risk environments) and, for adoption data, selective placement. Nonetheless, this simplified model provides a good starting point for comparing results from different studies.

### Genetic Influences on Alcoholism Risk

For individual studies, Heath and colleagues (in press) estimated the proportions of variability in alcoholism liability explained by genetic and family environmental influences. Several results are striking.

First, our analysis does not support the common belief that genetic influences on alcoholism risk may be more important in men than in women.

Second, two studies appear to be outliers, producing results at variance with the general trend. The estimate of the shared environmental contribution to alcoholism risk from the Kaij (1960) study is much greater than in all other studies. The reasons for this are unknown, although it is possible that in the work by Kaij some registrations were accidentally overlooked. However, once one twin from a pair was identified with a registration, the records were searched more thoroughly to determine whether the co-twin also had been registered. Cadoret’s study of four adoption agencies (Cadoret 1994; Cadoret et al. 1995) has yielded a high estimate of the genetic contribution to variability in alcoholism risk, which does not differ significantly from a probability of 100 percent (i.e., complete heritability). Further inquiry is needed to determine the cause of this result.

Third, the combined genetic variability and family environmental contributions to alcoholism risk in the Iowa CFS and LSS studies total 100 percent, implying that nonshared environmental effects have no impact on alcoholism risk. This result seems implausible. As noted previously, alcoholism in adoptive families could include drinking problems that were caused by the behavior of the adoptee. Thus, the contributions of environmental and genetic variability would not be independent of each other. With the exception of the two outlier studies, in the remaining studies, nonshared environmental influences account for at least 30 percent of the variation in alcoholism risk.

In a meta-analysis, we have jointly analyzed data from all studies already mentioned, except the two outliers. Meta-analysis is used to combine data from many different studies to improve the ability to detect small effects by improving the preciseness of estimates. Estimates of genetic and environmental effects did not appear to vary significantly within the group of U.S. studies or the group of Scandinavian studies. In analyzing the U.S. data, estimates for the contributions of the family environment obtained from the adoption data (i.e., based on the Cadoret LSS and CFS samples) and from the twin data were considered separately because of concerns about the measure of the adoptive family’s environmental contribution to alcoholism mentioned previously.

Within both the U.S. and Scandinavian studies, no significant gender differences were found in the genetic contribution to alcoholism risk. In the Scandinavian data, genetic factors appear to be more important in women than in men (a pattern that is seen in both the Swedish adoption and Swedish twin studies), but no statistically significant difference exists. Based on the U.S. data, genetic effects account for approximately 60 percent of the variance in alcoholism risk in both men and women, and the twin data suggest that there is no significant effect of family environment. The U.S. adoption data suggest that the adoptees’ family environments may account for one-third of the variance. The Scandinavian data yield a lower estimate for the importance of genetic influences (i.e., 39 percent) and a modest but significant estimate for family environmental influences (i.e., 15 percent). This difference between the U.S. and Scandinavian data appears to be explained largely by differences in Scandinavian males, with estimates for Scandinavian women being close to those for U.S. men and women.

## Conclusions

The reanalysis reviewed here has confirmed the consistency of the evidence for an important genetic influence on alcoholism risk from both twin and adoption studies. The genetic influence on alcoholism in women appears as strong as that in men. Many studies that followed children of alcoholics prospectively to identify precursors of alcoholism risk have focused on sons of alcoholics, assuming a stronger genetic influence in men than in women (for further discussion of markers, see the article by Anthenelli and Tabakoff, pp. 176–181). This assumption does not appear to be supported by the data, at least for the definitions of alcoholism analyzed here.

Second, estimates from Heath and colleagues (in press) have been remarkably consistent across groups born in different time periods—that is, whether one compares U.S. male twins born in the 1920’s who served in World War II or U.S. adoptees born in the 1940’s, 1950’s, and 1960’s, or whether one compares Swedish female adoptees born from 1930 to 1949 and Swedish female twins born as late as 1967. Given the changes that occur over time in the levels of alcohol consumption and the rates of alcohol problems, it is somewhat surprising that the importance of genetic factors has not changed, although a similar finding has been observed for genetic influences on smoking behavior (Heath et al. 1993; Heath and Madden 1995). It could have been anticipated that increasing exposure to alcohol would make genetic factors become more important.

Third, results have been remarkably consistent despite major differences between samples in the methods used to diagnose alcohol problems, ranging from hospitalization or other treatment records and Swedish Temperance Board registrations to direct interview assessments, which, in community samples, include a relatively high proportion of “mild” cases. This pattern would only be expected if the same risk factors, genetic or environmental, operate across the entire spectrum of alcohol problems, from mild to severe. Kendler and colleagues (1992) noted this finding in women.

Fourth, an apparent cross-cultural difference in the importance of genetic influences on alcoholism risk is evident from these data, which, if confirmed, would be an important example of gene-environment interaction. The data raise the possibility that, at least in men, genetic factors may have a reduced impact on alcoholism risk in some environments (e.g., Scandinavian) compared with other environments (e.g., U.S.). However, this could be an artifact of differences in research methodology, as no studies led by the same investigators and using a common research methodology have been conducted in both Scandinavia and the United States.

Some important limitations should be noted. Although studies now include Hispanic, African-American, Asian, and other minority groups, most earlier studies predominantly used samples of European ancestry. The generalizability of findings in populations of non-European ancestry remains to be determined.

This article has focused on questions about the relative importance of genetic and environmental influences on alcoholism. However, such questions are only a starting point for behavioral genetic research on alcoholism. Understanding more about how genes and environment act, co-act, and interact to determine differences in alcoholism risk remains a key goal of ongoing twin and adoption studies. Meanwhile, the evidence from twin and adoption studies has provided researchers with the impetus to investigate other methods of genetic alcoholism research, such as molecular genetics studies and the development of animal models. Together, these endeavors will continue to shed light on the genetic contribution to alcoholism.

## Footnotes

1 A risk ratio is a measure of association between two variables. Here, the control adoptees have been assigned a risk of 1 because they are the group against which the other groups in the study are measured. A risk ratio of 3.6 for adopted-away sons of alcoholics thus means that that group is 3.6 times as likely as the control adoptees to become alcoholic.

2 A genetic overlap appears to exist between alcoholism and antisocial behavior.

3 A co-twin is one member of a pair of twins.

4 An extension of that study, which includes male same-sex pairs as well as opposite-sex pairs, is now in progress.

5 This survey was designed to study the co-occurrence of alcohol and other drug use disorders with psychiatric disorders in the United States.

6 However, results reached significance if the diagnostic criteria were either narrowed (to require withdrawal symptoms) or broadened (to include problem drinking).
